# Racial and Ethnic Disparities Among Participants in Precision Oncology Clinical Studies

**DOI:** 10.1001/jamanetworkopen.2021.33205

**Published:** 2021-11-08

**Authors:** Christopher M. Aldrighetti, Andrzej Niemierko, Eliezer Van Allen, Henning Willers, Sophia C. Kamran

**Affiliations:** 1 Department of Radiation Oncology, Massachusetts General Hospital, Harvard Medical School, Boston; 2 Dana-Farber Cancer Institute, Boston, Massachusetts; 3 Broad Institute of Harvard and MIT, Cambridge, Massachusetts; 4 Center for Cancer Precision Medicine, Dana-Farber Cancer Institute, Boston, Massachusetts

## Abstract

**Importance:**

Precision oncology is revolutionizing cancer care, allowing for personalized treatments to improve outcomes. Cancer research has benefitted from well-designed studies incorporating precision medicine objectives, but it is unclear if these studies are representative of the diverse cancer population.

**Objective:**

To evaluate racial and ethnic representation in breast, prostate, lung, and colorectal cancer studies incorporating precision oncology objectives in the Clinicaltrials.gov registry and compare with the incidence of these cancer types in racial and ethnic minority groups in the US population.

**Design, Setting, and Participants:**

This cross-sectional study identified US-based breast, prostate, lung, and colorectal cancer studies incorporating precision oncology objectives for reporting of race and ethnicity. The Surveillance, Epidemiology, and End Results and US Census databases were used to determine cancer incidence by race and ethnicity, linked with cancer type and median year of enrollment for each trial. Data were collected and analyzed between December 2020 and April 2021.

**Main Outcomes and Measures:**

The expected number of participants per study by each racial and ethnic group was calculated based on the corresponding US-based proportion. Under- and overrepresentation was defined as the ratio of the actual number of enrolled cases to the expected number of cases for each trial by cancer type. Ratios above 1 indicated overrepresentation while a ratio below 1 indicated underrepresentation. Random-effects meta-analysis of representation ratios of individual trials was performed to weigh each individual study.

**Results:**

Of 93 studies encompassing 5867 enrollees with race and ethnicity data; 4826 participants (82.3%) were non-Hispanic White, 587 (10.0%) were Black, and 238 (4.1%) were Asian. Per observed-to-expected ratios, White participants were overrepresented in all studies, with a ratio of 1.35 (95% CI, 1.30-1.37), as well as Asian participants, with a ratio of 1.46 (95% CI, 1.28-1.66), while Black participants (ratio, 0.49; 95% CI, 0.45-0.54), Hispanic participants (ratio, 0.24; 95% CI, 0.20-0.28), and American Indian and Alaskan Native participants (ratio, 0.43; 95% CI, 0.24-0.78) were underrepresented. By individual cancer site, White participants were consistently overrepresented in all studies, while Black and Hispanic participants were underrepresented.

**Conclusions and Relevance:**

This analysis found that precision oncology studies for breast, lung, prostate, and colorectal cancers vastly underrepresent racial and ethnic minority populations relative to their cancer incidence in the US population. It is imperative to increase diversity among enrollees so that all individuals may benefit from cancer research breakthroughs and personalized treatments.

## Key Points

**Question:**

What is the representation of racial and ethnic minority populations in studies incorporating precision oncology objectives in the US?

**Findings:**

This cross-sectional analysis evaluates breast, prostate, lung, and colorectal cancer studies in the Clinicaltrials.gov registry with precision medicine objectives and reporting race and ethnicity—a total of 93 studies with 5867 total enrollees. An underrepresentation of minority racial groups and an overrepresentation of non-Hispanic White participants relative to their incidence in the US cancer population was found in precision oncology studies.

**Meaning:**

These findings demonstrate an urgent need to increase enrollment of participants from diverse racial and ethnic backgrounds onto precision oncology studies, so that meaningful precision data can be collected and stratified to traditionally underrepresented participants.

## Teaser

This cross-sectional study evaluates the representation of racial and ethnic minority groups in breast, prostate, lung, and colorectal cancer studies with a focus on precision medicine objectives.

## Introduction

Precision medicine has revolutionized oncology in the past 2 decades and is expected to continue to transform cancer management. The principle of personalized cancer therapy incorporates a multi-omic (eg, genomic, transcriptomic, metabolomic, proteomic, epigenetic) approach along with other tumor- and patient-specific factors. The identification of biomarkers facilitates stratifying patients and guiding treatment individualization, leading to improved outcomes.^1^ Much biomarker discovery and translational research that contribute to personalized treatments come from well-designed studies that incorporate precision medicine principles. Additionally, knowledge learned from studies that are designed to collect translational data can be used as part of discovery for predictive and prognostic biomarkers, which then can be applied to personalized therapeutic regimens.

Several targetable biomarkers have been found to have racial and ethnic differences in incidence for different cancer types such as epidermal growth factor receptor variants in lung cancer associating with East Asian ethnicity^2^ as well as Native American ancestry.^3^ Separately, growing evidence suggests underlying tumor genomic differences between African American vs White men with prostate cancer.^4,5^ This information can lead to additional study and discovery as well as novel treatment options for those who harbor the specific tumor alteration or biomarker. However, the use of race and ethnicity in such analyses has multiple limitations. Race and ethnicity must be understood as social constructs. We also recognize that the traditionally broad racial and ethnic categories do not capture the heterogeneity that exists within each group.^6^ Consequently, even the aforementioned findings should be further explained and contextualized in terms of other social determinants of health, such as health insurance status, zip code, socioeconomic status, environment, and other characteristics. The intersection of social, environmental, and genomic and/or biologic factors and the resultant influence on disease is poorly understood, with calls to action to capture these data points more robustly in all future precision medicine studies to evaluate and understand these interactions.^7,8^

The use of genetic ancestry has been lauded as a tool that can better differentiate populations in the study of biology and genomics.^8,9,10^ However, genetic ancestry data are not yet readily available for clinical practice or for use in clinical studies, nor have that data been historically captured.^11^ The historical static groupings of individuals by race and ethnicity with regards to reported health data over time have led to the acceptance and use of these categories in biomedical research.^7^ Stratification by various racial and ethnic groupings has identified several important differences in disease risk as well as response to certain treatments.^11^ In addition, the historical use of race and ethnicity as currently defined to differentiate patients in the study of disease has led to the emergence and recognition of medically underserved and underrepresented minority populations.^12^ Thus, although imperfect, understanding disparities by racial and ethnic groupings is of value, particularly pertaining to the study of precision medicine, as it is important to understand from which populations we are collecting our biologic and biomarker data for use in the general cancer population.

Despite rapid diversification of the US cancer population,^13,14^ enrollment of a diverse patient population into cancer clinical trials lags behind, including for racial and ethnic minority groups.^15,16,17^ As more clinical studies specifically incorporate precision oncology principles into their design, it is unknown whether ethnic and racial minority populations are adequately represented, an important consideration when one critical question is whether precision oncology discoveries in clinical trials are broadly applicable to the general cancer population. Is precision oncology research missing crucial findings that could be benefiting traditionally marginalized groups, thus further widening inequality and contributing to disparities in cancer care? Moreover, any identifiable differences between groups in a single or across a collection of studies must be further evaluated, particularly if personalization of a therapeutic is identified for 1 group vs another, as sample sizes would be too small to gather appropriate safety data. Both prespecified analyses in postapproval settings as well as the use of real-world study would be necessary to interrogate safety and efficacy data.^18^ However, the opportunity to look for differences between various racial and ethnic groupings requires a concerted effort towards recruitment of underserved populations to these studies in their initial stages. Therefore, we sought to understand how well precision oncology studies are representative of the diverse US cancer population. Focusing on the top causes of new cancer cases in the US (ie, breast, colorectal, lung, and prostate cancers),^19^ we evaluated clinical studies with precision medicine objectives for their reporting of race and ethnicity, and asked whether these study demographics were representative of the diverse US cancer population.

## Methods

In this cross-sectional study, the Clinicaltrials.gov registry was queried for completed US-based clinical studies by cancer type (breast, colorectal, lung, and prostate) with results incorporating precision medicine objectives based on a set of precision oncology search terms: *immunohistochemistry*, *histopathologic*, *DNA*, *RNA*, *DNA sequencing*, *RNA sequencing*, *sequencing*, *proteomics*, *tumor mutational burden*, *tumor biomarkers*, *biomarkers*, *tumor analysis*, *mutation testing*, *mutational analysis*, *microarray*, *whole exome*, *molecular analysis*, *genomics*, *genetics*, *gene expression*, *expression*, *signatures*, *genetic testing*, and *prognostic testing*. Studies were excluded if they included site locations outside the US, did not include diagnosis of cancer (eg, precancerous), or did not incorporate precision oncology measures. Eligible studies were evaluated for reporting of racial and ethnic demographic data. Additional data gathered included type of clinical study (eg, phase 1) and type of funding (National Institutes of Health [NIH] vs other). Participant demographics were obtained from Clinicaltrials.gov if present and corroborated with primary report journal articles or abstract and/or presentation if unpublished (per availability). Racial and ethnic groupings were mutually exclusive as follows: American Indian/Alaskan Native, Asian, Black, Hispanic, and Non-Hispanic White participants. Participants with race and ethnicity not reported or unknown were removed from the analysis. Overall, these individuals made up less than 3% of the total enrollees per each cancer type (eg, overall, 126 of 5993 participants [2.1%]). Clinicaltrials.gov was queried in December 2020 with an update of the query and analysis in April 2021.

Cancer incidence by race and ethnicity within the US cancer population, correlated with cancer type and median year of enrollment for each study, was collected from the National Cancer Institute Surveillance, Epidemiology, and End Result (SEER) database.^20^ Age-standardized incidence rates adjusted to the 2000 standard US population by race and ethnicity were used to calculate cancer cases as a measure of proportion of cancer burden by race and ethnicity using US Census Bureau tables.^21,22^ This study was determined to be exempt from human participant research guidelines because it was a secondary analysis of publicly available published reports and data by the Mass General Brigham institutional review board. We followed the Strengthening the Reporting of Observational Studies in Epidemiology (STROBE) reporting guideline for cross-sectional studies and the Preferred Reporting Items for Systematic Reviews and Meta-analyses (PRISMA) reporting guideline.

### Statistical Analysis

For each trial, the expected number of participants of each of the 5 racial and ethnic groups was calculated by multiplying the total number of participants in a trial with the US-based proportion of each group for a given cancer type and median year of enrollment. We defined underrepresentation and overrepresentation as the ratio of the actual number of enrolled cases and the expected number of cases for each trial and per cancer type, with 95% exact binomial CIs estimated. A ratio greater than 1 indicated overrepresentation, while a ratio smaller than 1 indicated underrepresentation. Since the expected number of participants for groups other than non-Hispanic White is small, especially for smaller trials, we calculated totals of expected to enrolled ratios in all precision studies and per cancer type.

Separately, we also performed random-effects meta-analysis of underrepresentation and overrepresentation ratios of individual trials (still relative to the US-based proportion of racial and ethnic groups for a given cancer type and median year of enrollment), with the exclusion of trials with no enrolled participants of a given racial or ethnic group. Therefore, the weighted average of underrepresentation and overrepresentation effect sizes from the meta-analysis might be biased for minority populations with small numbers of enrolled participants (eg, Asian). Results are presented using forest plots (eFigures 2-5 in the Supplement) showing study-specific effect sizes, and overall effect size for each cancer type and racial and ethnic group, with respective 95% CIs. Test statistics for the pooled data and estimates for between-trial heterogeneity statistics are also shown. Data were analyzed using Stata version 16 (StataCorp). To evaluate funding source and racial and ethnic reporting or distribution, Pearson χ^2^ and 2-sample Wilcoxon rank-sum tests were performed, respectively; *P* ≤ .05 was considered significant in 2-sided tests.

## Results

**Figure 1.**
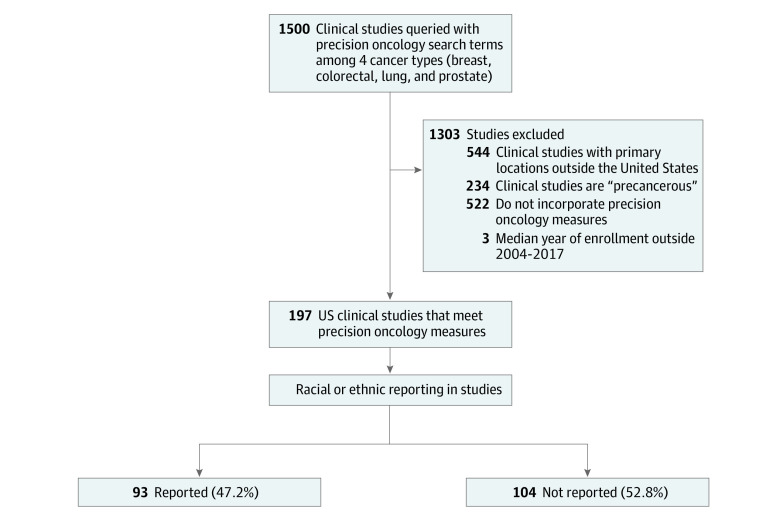
Study Schema and Race and Ethnicity Reporting in Precision Oncology Clinical Studies

Overall, 1500 studies were queried among the 4 cancer types and 1303 were excluded (Figure 1). There were 197 clinical studies that met precision oncology measures that were assessed for race and ethnicity analysis. When evaluating for racial and ethnic reporting, 93 studies (47.2%) had appropriate data, while 104 studies (52.8%) did not report any information about race or ethnicity. Over time, the proportion of precision oncology studies reporting race and ethnicity has increased (eFigure 1 in the Supplement). The median enrollment year for the included studies spanned from 2004 to 2017. Characteristics surrounding study phase and funding for studies (both included and excluded on basis of racial and ethnic group reporting) are shown in Table 1. There was no statistical association between source of funding (NIH vs other) and reporting of race and ethnicity.

**Table 1.** Precision Oncology Study Funding and Study Phase Information

Cancer type	Studies, No. (%)							
	Lung		Colorectal		Breast		Prostate	
	Included (n = 26)	Excluded (n = 32)^a^	Included (n = 14)	Excluded (n = 17)^a^	Included (n = 38)	Excluded (n = 31)^a^	Included (n = 15)	Excluded (n = 24)^a^
Study phase								
Phase 1	1 (4)	2 (6)	1 (7)	2 (12)	3 (8)	3 (10)	2 (13)	1 (4)
Phase 1 | Phase 2	4 (15)	5 (16)	0	2 (12)	6 (16)	5 (16)	3 (20)	2 (8)
Phase 2	19 (73)	24 (75)	11 (79)	13 (76)	23 (60)	19 (61)	8 (53)	18 (76)
Phase 3	1 (4)	0	1 (7)	0	1 (3)	0	1 (7)	1 (4)
Other	1 (4)	1 (3)	1 (7)	0	5 (13)	4 (13)	1 (7)	2 (8)
Funder								
NIH	16 (62)	17 (53)	8 (57)	9 (53)	14 (37)	15 (48)	9 (60)	10 (42)
Other	10 (38)	15 (47)	6 (43)	8 (47)	24 (63)	16 (52)	6 (40)	14 (58)

Abbreviation: NIH, National Institutes of Health.

^a^ Excluded on basis of no racial and ethnic reporting.

Of the 93 studies encompassing 5867 enrollees with recorded race and ethnicity data, most participants were non-Hispanic White (4826 [82.3%]), followed by Black participants (587 [10.0%]), Asian participants (238 [4.1%]), Hispanic participants (200 [3.4%]), with the lowest representation of American Indian/Alaskan Native participants (16 [0.3%]). By cancer type, lung cancer studies had the highest proportion of White (2054 of 2399 [85.6%]) and Asian (116 [4.8%]) participants, and the lowest proportion of Black (199 [8.3%]), Hispanic (26 [1.1%]), and American Indian/Alaskan Native (4 [0.2%]) participants. Colorectal cancer studies had the highest proportion of Hispanic participants (59 of 999 [5.9%]). Prostate cancer studies had the highest proportion of Black participants (67 of 596 [11.2%]) and American Indian/Alaskan Native participants (5 of 596 [0.8%]), and the lowest proportion of Asian participants (6 of 596 [1.0%]). When evaluating racial and ethnic distribution by funding type, Asian individuals were statistically overrepresented on studies with NIH funding.

**Figure 2.**
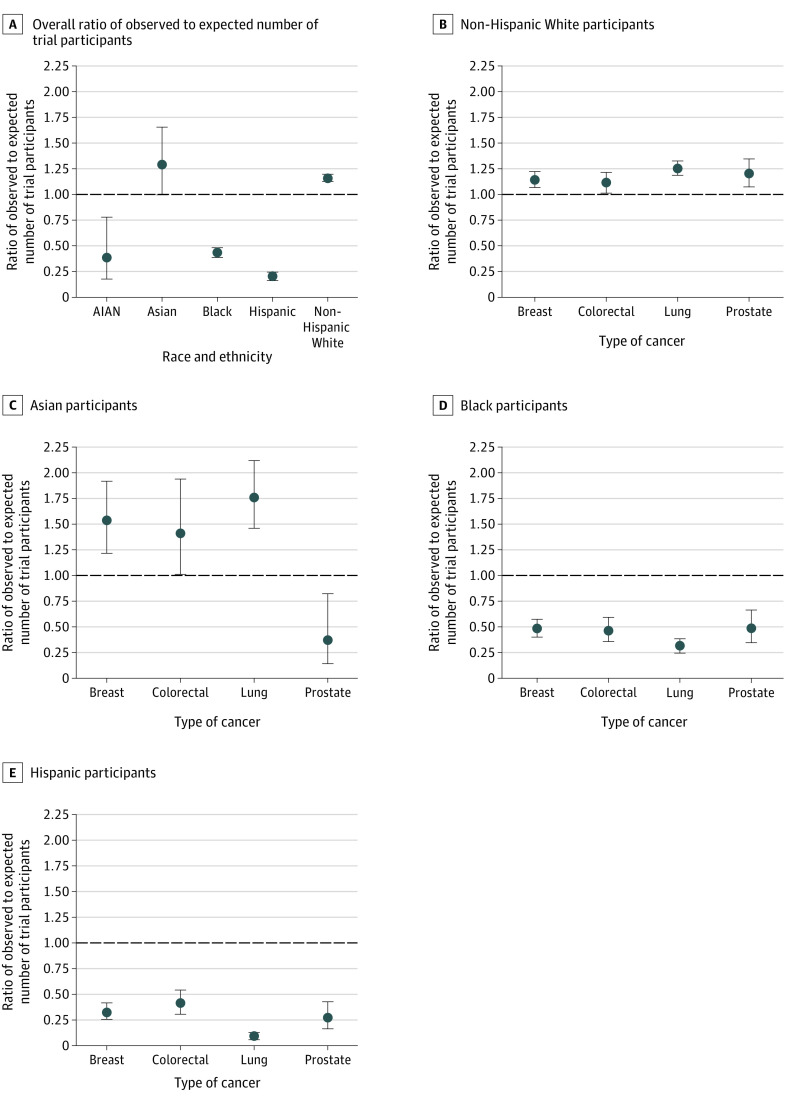
Racial and Ethnic Disparities in Precision Oncology Trials Compared to US Cancer Population Dots represent ratio; whiskers, 95% CI. Ratios below the dotted line indicate underrepresentation in oncology study enrollment.

When evaluating observed-to-expected ratios by race and ethnicity in all precision oncology studies, White and Asian participants were overrepresented with a ratio of 1.35 (95% CI, 1.30-1.37; 4826 observed vs 3582 expected) and 1.46 (95% CI, 1.28-1.66; 233 observed vs 160 expected), respectively (Figure 2). The other racial groups were substantially underrepresented: Black participants with a ratio of 0.49 (95% CI, 0.45-0.53; 587 observed vs 1181 expected), Hispanic participants with a 0.24 ratio (95% CI, 0.20-0.27; 200 observed vs 845 expected), and American Indian/Alaskan Native participants with a 0.43 ratio (95% CI, 0.25-0.70; 16 observed vs 37 expected). When evaluating ratios by cancer type, White participants were consistently overrepresented, while Asian participants were overrepresented in lung, colorectal, and breast cancer studies. Black and Hispanic participants were underrepresented in all cancer type studies. American Indian/Alaskan Native participants are not shown by cancer type because they are represented in very small numbers.

In the meta-analysis, which weighs individual studies, White participants remained overrepresented overall with a ratio of 1.34 (95% CI, 1.29-1.39) and by cancer type (Table 2; eFigure 2 in the Supplement). Overrepresentation ranged from 22% (colorectal cancer) to 40% (prostate and lung cancer). Asian participants were overrepresented in all studies with a ratio of 1.89 (95% CI, 1.46-2.32) (eFigure 3 in the Supplement). Cancer type–specific overrepresentation ranged from 29% (colorectal cancer) to 196% (lung cancer). Black participants remained underrepresented among all cancer types with a ratio of 0.51 (95% CI, 0.43-0.60) (eFigure 4 in the Supplement). Among each cancer type, underrepresentation ranged from 38% (breast cancer) to 68% (lung cancer). Similarly, Hispanic participants were underrepresented among all cancer types per meta-analysis (ratio, 0.51; 95% CI, 0.37-0.66) (eFigure 5 in the Supplement). Cancer type–specific underrepresentation ranged from 36% (breast cancer) to 69% (lung cancer). Numbers for American Indian/Alaskan Native participants were too small for accurate meta-analysis performance.

**Table 2.** Meta-analysis Ratios of Individual Precision Oncology Studies by Cancer Type

	Participants, ratio (95% CI)^a^			
	Non-Hispanic White	Asian	Black	Hispanic
Breast	1.32 (1.23 to 1.41)	1.95 (1.26 to 2.64)	0.62 (0.44 to 0.80)	0.64 (0.37 to 0.90)
Colorectal	1.22 (1.06 to 1.38)	1.29 (0.69 to 1.89)	0.60 (0.39 to 0.81)	0.61 (0.12 to 1.10)
Lung	1.40 (1.32 to 1.47)	2.96 (1.51 to 4.42)	0.32 (0.24 to 0.40)	0.31 (0.11 to 0.52)
Prostate	1.40 (1.29 to 1.52)	1.45 (−0.81 to 3.70)	0.58 (0.30 to 0.85)	0.33 (0.18 to 0.47)
Overall	1.34 (1.29 to 1.39)	1.89 (1.46 to 2.32)	0.51 (0.43 to 0.60)	0.51 (0.37 to 0.66)

^a^ Numbers for American Indian/Alaskan Native individuals were too small for accurate meta-analysis performance.

## Discussion

The underrepresentation of racial and ethnic minority populations in clinical trials relative to their cancer burden in the US has been previously reported.^13,15^ There is an urgent need to increase equitable recruitment of diverse participants to cancer clinical trials, as well as to understand racial and ethnic differences in treatment outcomes. However, the field of oncology has been moving toward personalized treatments based on presence of tumor molecular markers as well as genetic and genomic variation. Despite this, there is limited knowledge of underlying cancer biology in racial and ethnic minority groups. Current personalized treatments that are broadly generalized to all individuals based on the presence or absence of a biomarker without fully studying the implications of such treatment in racial and ethnic minority populations may not be appropriate. Even if biological differences are discovered that are unique to certain racial or ethnic minority subgroups, these must be further validated and evaluated for safety and efficacy. Similarly, large biorepositories from which to study precision omics are disproportionately dominated by individuals of European ancestry.^10,23,24^ This is a well-documented issue with genomewide association studies,^10^ particularly for cancer research, with European-ancestry groups making up 96% of participants, while 0.11%, 0%, and 0.5% of subjects are from African, African American and Afro-Caribbean, and Hispanic and Latin American groups in studies at the initial discovery phase in 2019,^25^ prompting the call for more inclusive samples and inclusive research communities. Thus, it is insufficient to take our current understanding of tumor biomarkers and biology and generalize findings to the entire diverse cancer population. Although race and ethnicity have been used historically to stratify populations, to fully understand any biological findings that differ by these social constructs, it is critical that future studies prospectively capture additional data that accounts for social determinants that influence disease, as well as data incorporating the use of genetic ancestry.^8,9,11^

As contemporary clinical studies specifically incorporate precision oncology principles, it is unknown whether racial and ethnic minority populations are adequately represented overall and by cancer type relative to their cancer incidence in the US cancer population. By focusing on the most common cancer types in the US (breast, prostate, lung, and colorectal cancers), we found that most precision oncology studies do not report the race and ethnicity of participants. Of those studies with racial and ethnic reporting, we demonstrated an underrepresentation of minority racial groups and an overrepresentation of Non-Hispanic White participants in precision oncology studies. Black and Hispanic participants were the most underrepresented on lung cancer studies by 68% and 69% per meta-analysis, respectively. On the other hand, Asian individuals were overrepresented in lung cancer studies by 196%, consistent with the prominent role of targetable oncogene drivers in this population. There is likely underlying genetic susceptibility for these alterations among the Asian population in addition to the epidemiologic association with nonsmoking status.^26,27,28,29^ Yet, data now demonstrate differential biology (as it relates to somatic driver mutations) among Hispanic participants with lung adenocarcinoma, underscoring the importance of their increased representation on precision oncology lung trials, as well as capturing of social determinants of disease to analyze these associations further. Separately, Hispanic individuals also had poor representation in prostate cancer studies (by 67%). Overall, our findings data highlight the need to increase enrollment of these groups into future clinical studies. All individuals deserve to benefit from cancer research breakthroughs and deep understanding of the underlying tumor biology not only in the context of race and ethnicity, but in the context of social determinants of health as well.

There is a long history surrounding underrepresentation of medically underserved participants on trials.^12,18^ There are multiple factors at play, including medical mistrust.^30^ Prior work has found that individuals from underrepresented racial and ethnic groups’ willingness to participate in research stems from perceived trustworthiness of the researchers, the institutions conducting the research, and the information provided about the applicable research studies.^31^ There may be more mistrust surrounding genetic and biological studies—for example, genetic counseling participation is low among Black women for *BRCA1/2* testing.^32^ This finding was exacerbated among women with higher rates of medical mistrust,^32^ fear of discrimination by insurance companies if considered high-risk,^33^ and fear of carrying the mutation.^34^ A targeted study evaluating African American and Hispanic perspectives on the prospect of precision medicine found that, although groups believed that precision medicine can improve health outcomes, both groups were concerned that current barriers to health care would prevent their communities from benefiting from precision medicine.^35^

Multiple strategies must be implemented to increase diverse enrollment onto precision oncology trials, including patient navigation programs, patient education about multi-omic studies, patient education about genetics and genetic counseling, unique trial designs, education for all researchers or members involved to combat bias, and other educational resources to address barriers to enrollment for trial participation. More research is needed to fully understand all barriers and fears surrounding research recruitment. An increase in racial and ethnic diversity among scientists, biomedical researchers, physicians, and clinical trialists can help to increase trust among patients from minoritized groups.^11,36^ Finally, precision oncology studies should increase the reporting of race and ethnicity of participants so that improvements in diversity and representation can be observed over time, and this analysis can serve as a benchmark with which to compare future progress.

### Limitations

Limitations to this analysis include those associated with use of Clinicaltrials.gov. We queried studies with reported results, which could miss noncompliant and incompletely accrued studies. Our search criteria could have missed slow-accruing precision oncology studies, or those that remained open after reaching their primary end point. We may be missing crucial precision oncology studies that were not identified with the search terms used, as well as studies that did not prespecify these analyses and were instead performed post hoc. Second, no information about race and ethnicity assessment was provided—it is important to emphasize that the racial and ethnic categories as reported do not capture the cultural heterogeneity within these groups. Third, there is no information on multiracial or multiethnic patients, which fails to capture patients who might identify as more than 1 racial or ethnic group. We recognize that this is imperfect, particularly given that in the latest 2020 US Census, over 33.8 million Americans identified themselves as being multiracial, resulting in an increase of nearly 276% since the 2010 census.^14^ This highlights the opportunity for future studies or trials to capture these critical nuances in the future, along with demographic, social, environmental, and other determinants of health, to create fully annotated databases to enhance the study of biology and multi-omic research with a broader lens to understand the interaction of these multiple factors.

## Conclusions

Increased emphasis on equitable recruitment and enrollment for precision oncology studies is essential, as resulting discoveries are used to personalize treatments, and it is unclear whether current precision medicine breakthroughs can be broadly applicable to, or safe for, our diverse cancer population. We demonstrate that precision oncology studies for breast, lung, prostate, and colorectal cancers underrepresent racial and ethnic minority populations relative to their cancer incidence in the US population. All relevant stakeholders should implement strategies to increase diverse clinical study enrollment to address this disparity. A continued lack of diversity among trial enrollees may further leave behind undeserved minority populations in the era of precision oncology.

## Notes

Supplement. **eFigure 1.** Precision Oncology Studies by Race/Ethnicity Reporting Over Time **eFigure 2.** Meta-Analysis of NHW Participant Representation in Precision Oncology Trials Compared With US Cancer Incidence **eFigure 3.** Meta-Analysis of Asian Participant Representation in Precision Oncology Trials Compared With US Cancer Incidence **eFigure 4.** Meta-Analysis of Black Participant Representation in Precision Oncology Trials Compared With US Cancer Incidence **eFigure 5.** Meta-Analysis of Hispanic Participant Representation in Precision Oncology Trials Compared With US Cancer Incidence
