# Supplementary material for: Racial and Ethnic Disparities Among Participants in Precision Oncology Clinical Studies
Source: JAMA Netw Open. 2021 Nov 8;4(11):e2133205. doi: 10.1001/jamanetworkopen.2021.33205 (PMC8576580; doi:10.1001/jamanetworkopen.2021.33205)
Supplement: Supplement. — eFigure 1. Precision Oncology Studies by Race/Ethnicity Reporting Over Time eFigure 2. Meta-Analysis of NHW Participant Representation in Precision Oncology Trials Compared With US Cancer Incidence eFigure 3. Meta-Analysis of Asian Participant Representation in Precision Oncology Trials Compared With US Cancer Incidence eFigure 4. Meta-Analysis of Black Participant Representation in Precision Oncology Trials Compared With US Cancer Incidence eFigure 5. Meta-Analysis of Hispanic Participant Representation in Precision Oncology Trials Compared With US Cancer Incidence [file jamanetwopen-e2133205-s001.pdf]

Supplemental Online Content

Aldrighetti CM, Niemierko A, Van Allen E, Willers H, Kamran SC. Racial and ethnic disparities among participants in precision oncology clinical studies. *JAMA Netw Open.* 2021;4(11):e2133205. doi:10.1001/jamanetworkopen.2021.33205

eFigure 1. Precision Oncology Studies by Race/Ethnicity Reporting Over Time

eFigure 2. Meta-Analysis of NHW Participant Representation in Precision Oncology Trials Compared With US Cancer Incidence

eFigure 3. Meta-Analysis of Asian Participant Representation in Precision Oncology Trials Compared With US Cancer Incidence

eFigure 4. Meta-Analysis of Black Participant Representation in Precision Oncology Trials Compared With US Cancer Incidence

eFigure 5. Meta-Analysis of Hispanic Participant Representation in Precision Oncology Trials Compared With US Cancer Incidence

This supplemental material has been provided by the authors to give readers additional information about their work.

© 2021 Aldrighetti CM et al. *JAMA Network Open.*

eFigure 1. Precision oncology studies by race/ethnicity reporting over time

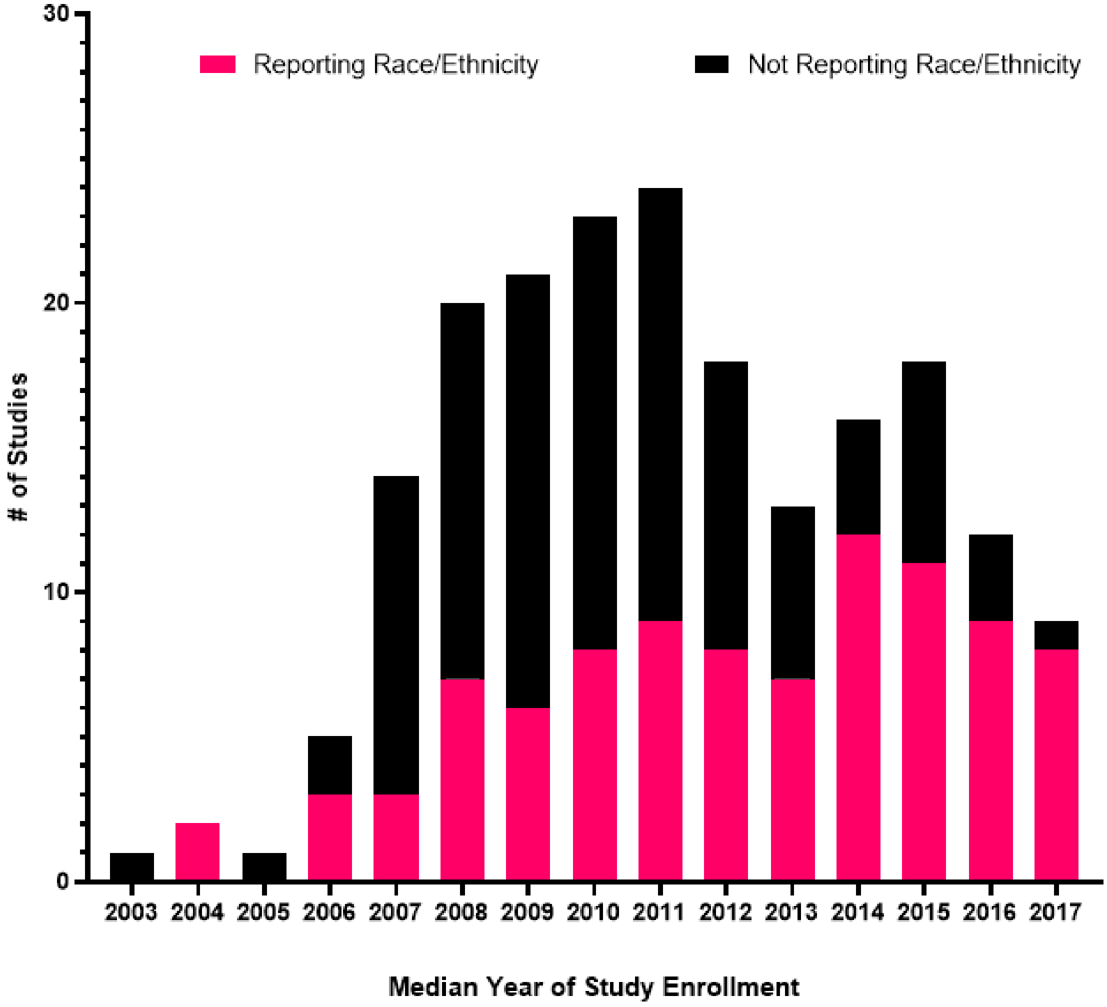

Studies are represented by median year of study enrollment. The proportion of studies reporting race/ethnicity has increased over time. The number of precision oncology studies is also increasing over time. The decline of studies after 2011 is likely due to fewer studies being “closed” at the time of this analysis in the more contemporary period.

© 2021 Aldrighetti CM et al. *JAMA Network Open.*

eFigure 2. Meta-Analysis of NHW participant representation in Precision Oncology Trials compared with US Cancer Incidence In this meta-analysis, which weighs individual studies, NHW participants were overrepresented overall with a ratio of 1.34 (95%CI 1.29-1.39), and by disease site. Overrepresentation ranged from 22% (colorectal cancer) to 40% (prostate and lung cancer).

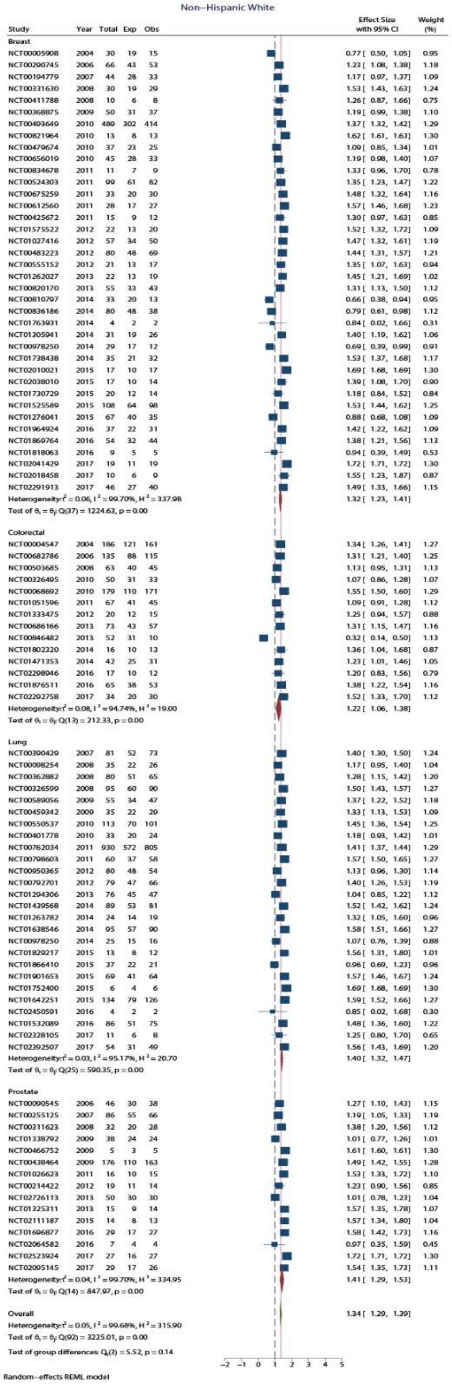

© 2021 Aldrighetti CM et al. *JAMA Network Open.*

eFigure 3. Meta-Analysis of Asian participant representation in Precision Oncology Trials compared with US Cancer Incidence In this meta-analysis, which weighs individual studies, Asian participants were overrepresented in all studies with a ratio of 1.89 (95%CI 1.46-2.32). Disease site-specific overrepresentation ranged from 29% (colorectal cancer) to 196% (lung cancer).

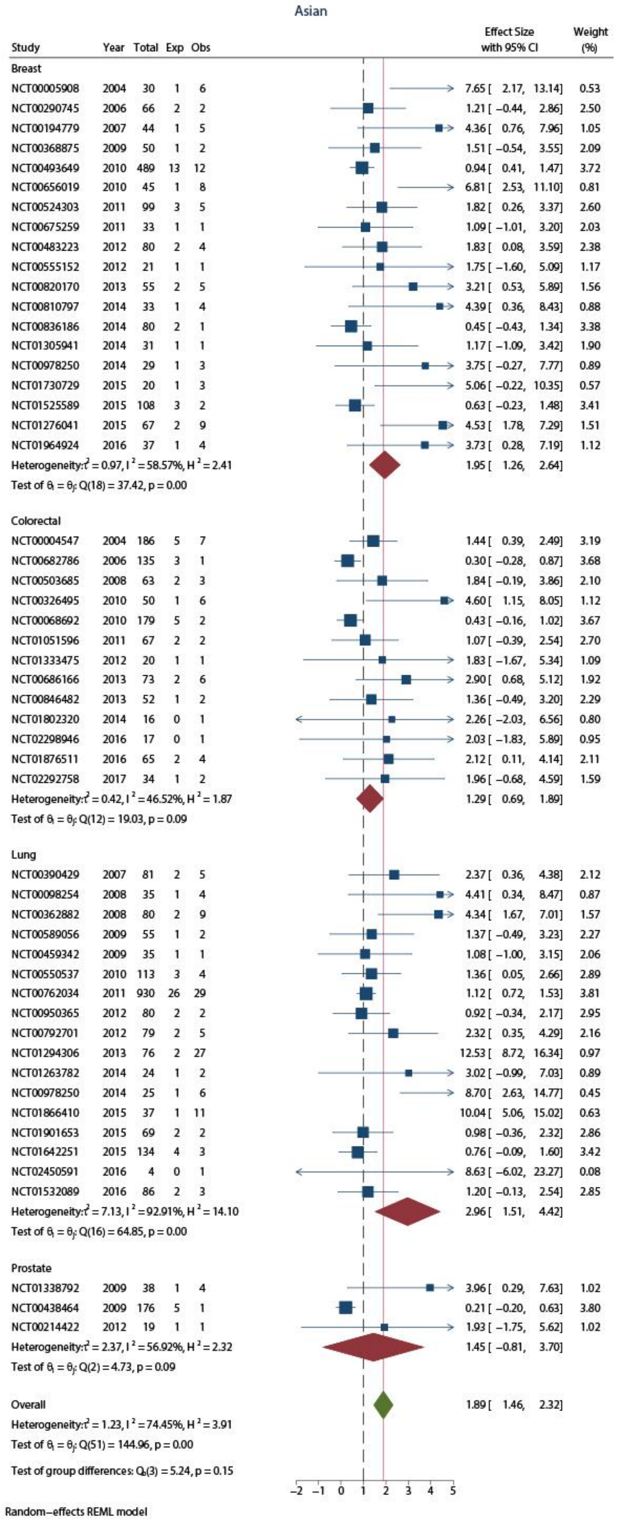

© 2021 Aldrighetti CM et al. *JAMA Network Open.*

eFigure 4. Meta-Analysis of Black participant representation in Precision Oncology Trials compared with US Cancer Incidence In this meta-analysis, which weighs individual studies, Black participants were underrepresented among all disease sites with a ratio of 0.51 (95%CI 0.43-0.60). Among each disease site, underrepresentation ranged from 38% (breast cancer) to 68% (lung cancer).

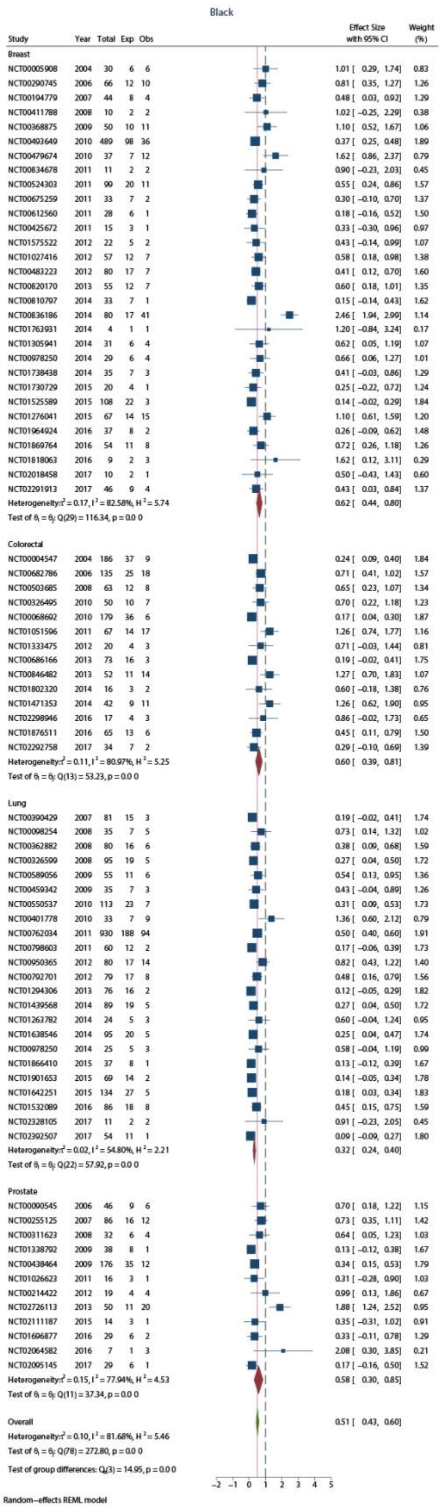

© 2021 Aldrighetti CM et al. *JAMA Network Open.*

eFigure 5. Meta-Analysis of Hispanic participant representation in Precision Oncology Trials compared with US Cancer Incidence In this meta-analysis, which weighs individual studies, Hispanic participants were underrepresented among all disease sites. Disease site-specific underrepresentation ranged from 36% (breast cancer) to 69% (lung cancer).

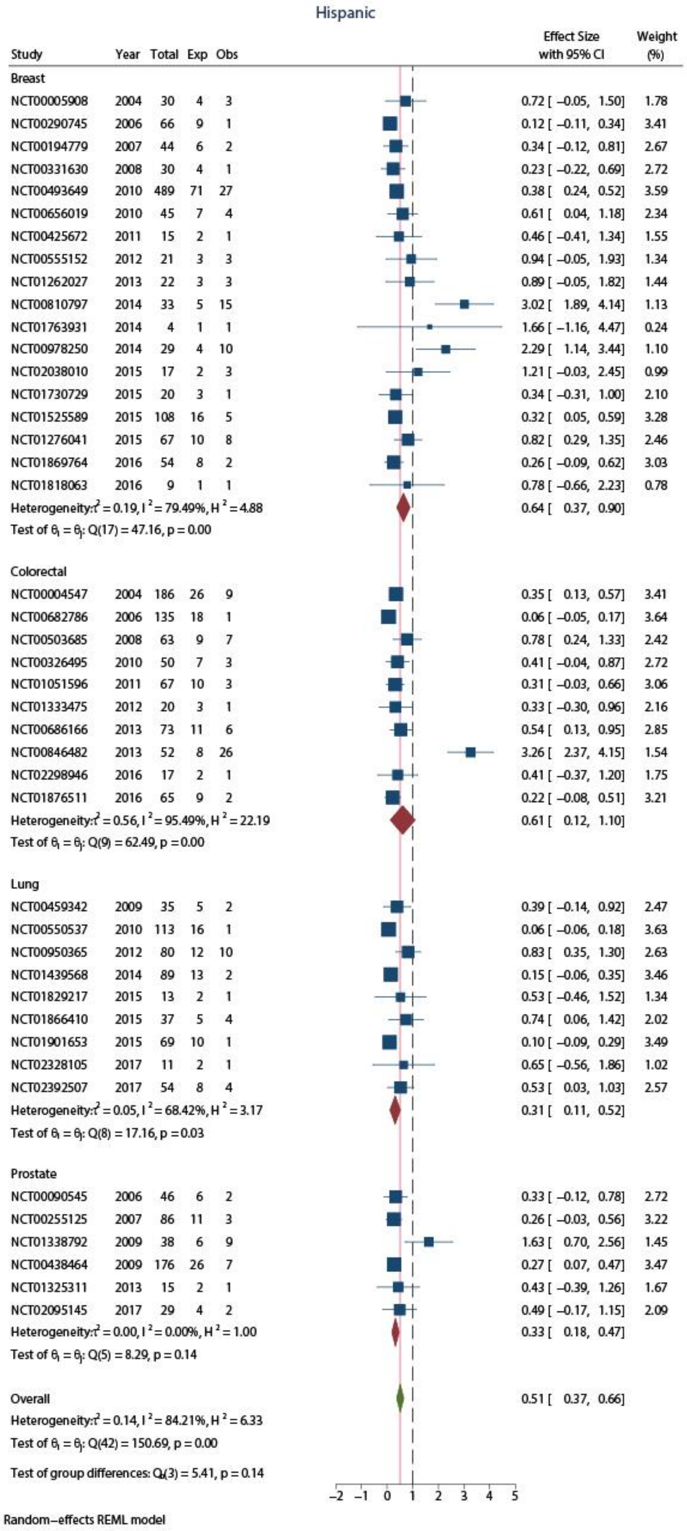

© 2021 Aldrighetti CM et al. *JAMA Network Open.*
